# H3K27me3 Signal in the *Cis* Regulatory Elements Reveals the Differentiation Potential of Progenitors During *Drosophila* Neuroglial Development

**DOI:** 10.1016/j.gpb.2018.12.009

**Published:** 2019-06-11

**Authors:** Xiaolong Chen, Youqiong Ye, Liang Gu, Jin Sun, Yanhua Du, Wen-Ju Liu, Wei Li, Xiaobai Zhang, Cizhong Jiang

**Affiliations:** 1 Institute of Translational Research, Tongji Hospital, School of Life Sciences and Technology, Shanghai Key Laboratory of Signaling and Disease Research, Tongji University, Shanghai 200092, China; 2 Tongji University Library, Tongji University, Shanghai 200092, China; 3 Research Center of Stem Cells and Ageing, Tsingtao Advanced Research Institute, Tongji University, Tsingtao 266071, China

**Keywords:** Nucleosome, Histone modification, Neural stem cell, Neuron, Glia

## Abstract

*Drosophila* neural development undergoes extensive chromatin remodeling and precise epigenetic regulation. However, the roles of chromatin remodeling in establishment and maintenance of cell identity during cell fate transition remain enigmatic. Here, we compared the changes in gene expression, as well as the dynamics of **nucleosome** positioning and key **histone modifications** between the four major neural cell types during *Drosophila* neural development. We find that the neural progenitors can be separated from the terminally differentiated cells based on their gene expression profiles, whereas nucleosome distribution in the flanking regions of transcription start sites fails to identify the relationships between the progenitors and the differentiated cells. H3K27me3 signal in promoters and enhancers can not only distinguish the progenitors from the differentiated cells but also identify the differentiation path of the **neural stem cells** (NSCs) to the intermediate progenitor cells to the glial cells. In contrast, H3K9ac signal fails to identify the differentiation path, although it activates distinct sets of genes with **neuron**-specific and **glia**-related functions during the differentiation of the NSCs into neurons and glia, respectively. Together, our study provides novel insights into the crucial roles of chromatin remodeling in determining cell type during *Drosophila* neural development.

## Introduction

Chromatin structure and state regulate many biological processes through controlling DNA accessibility. In general, chromatin structure is more open in the pluripotent stem cells than in the differentiated cells [1]. As the basic repeating unit of chromatin, nucleosome is important for establishing chromatin architecture. Therefore, nucleosome organization in the genome plays a critical role in modulating transcription, DNA replication, DNA repair, and other DNA template-based processes [2]. Nucleosome eviction and the resultant gene activation promote endodermal differentiation of mouse embryonic stem cells (mESCs) [3]. Nucleosome repositioning and variable nucleosome repeat length also have regulatory functions in cell lineage commitment [4]. Differential nucleosome occupancy between somatic cells and pluripotent stem cells often occurs in the regions containing binding sties for the pluripotency transcription factors OCT4, SOX2, *etc*. [5]. Thus, dynamic nucleosome positioning is a key regulatory mechanism underlying cell fate transition.

Similarly, histone modifications (HMs) are also involved in the regulation of many biological processes. HMs can serve a signal to recruit non-histone proteins to the target DNA sites [6]. Therefore, changes in HMs can lead to alterations in the chromatin state. Both global and gene-specific HM changes are required for mESC differentiation [7]. For instance, extensive HM changes occur and have impact on the differentiation of mESCs into neural progenitor cells [8]. In humans, H3K9ac signal recruits SOX2 and PAX6 to their target sites to facilitate the neuroectodermal differentiation from ESCs [9]. Interestingly, underacetylated H4 is essential for X-inactivation after mESC differentiation [10]. Profiling HMs across 16 developmental stages of hematopoietic differentiation in mice reveals the commitment path of each lineage without erroneously clustering intermediate progenitors of different cell lineages together [11]. Therefore, in addition to the pivotal regulatory role in the differentiation, HMs are able to reveal the differentiation potential of progeny.

Our previous study shows that during the early *Drosophila* embryonic development, formation of nucleosome-depleted regions (NDRs) in enhancers activates gene transcription for neural stem cells (NSCs) to differentiate into neurons. H3K27ac and H3K9ac changes in promoters, in coordination with their changes in enhancers, regulate gene expression during this process [12]. We also reveal chromatin remodeling patterns and the associated functions in the glial differentiation during early *Drosophila* embryonic development [13]. However, little is known about the epigenetic regulatory network of chromatin remodeling during neurogenesis from NSCs to intermediate progenitors to terminally differentiated neural cells. It also remains unclear which HM(s) can reveal the differentiation potential of intermediate progenitors.

To address the questions above, we integrated RNA-seq, MNase-seq, and HM ChIP-seq data of four *Drosophila* neural cell types: NSCs, intermediate neural progenitors with lineage commitment to the glial cells, as well as the neuronal cells and the glial cells. Our systematic epigenomic analysis reveals that H3K27me3 signal in the regulatory regions could establish the differentiation paths of NSCs and the relationships among NSCs, intermediate progenitors, and terminally differentiated neural cells.

## Results and discussion

### Gene expression profiles distinguish the neural progenitor cells and the differentiated cells

**Figure 1:**
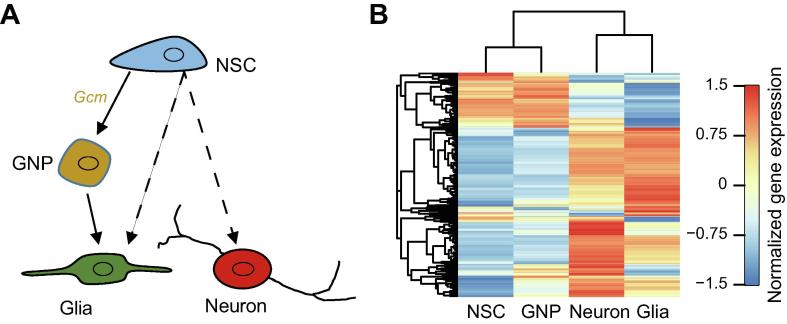
**Gene expression profiling separates the progenitor cells from the differentiated cells** **A.** Schematic diagram of NSCs differentiating to GNP cells, glia, and neurons. The solid and dashed lines indicate distinct differentiation paths. **B.** Unsupervised hierarchical clustering of gene expression normalized by Z-score fails to identify the differentiation path of NSCs to GNP cells to glia. NSC, neural stem cell; Gcm, glial cell missing; GNP, *Gcm*-expressed neural progenitor.

During *Drosophila* neuroglial development, upon the activation of the complicated gene regulatory network, the NSCs differentiate into glia and neurons, the two major types of neural cells (Figure 1A). As a transcription factor (TF), glial cell missing (Gcm) drives the glial differentiation [14]. The multipotent neural progenitor cells are committed to the glial cells when *Gcm* is expressed [15] (in this study, we name the *Gcm*-expressed neural progenitor cells as GNP cells).

Global gene expression profiles contain information regarding the transcriptional state of cells and can distinguish between cells of different types or states [16], [17]. Therefore, comparison of the gene expression profiles could allow us to identify the difference between these four neural cell types. Our results show that these cells are clustered into two groups: the progenitors (NSCs and GNP cells) and the terminally differentiated cells (neurons and glia) (Figure 1B). Nevertheless, the gene expression profiles failed to build the differentiation path of the NSCs to the GNP cells to the glial cells, indicating that gene expression may primarily reflect the pluripotency during *Drosophila* neuroglial development.

### Nucleosome organization in promoters fails to reveal the differentiation potential of intermediate progenitors

**Figure 2:**
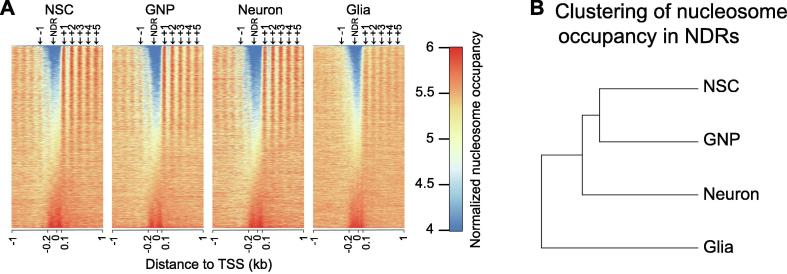
**Nucleosome organization around TSSs fails to reveal the neural progenitor–progeny relationships** **A.** Normalized nucleosome occupancy pattern around TSSs. Genes are ascendingly ordered by nucleosome occupancy in NDRs (ranging from 150 bp upstream to 50 bp downstream of TSSs). The typical arrangement at −1, NDR, +1, +2, +3 nucleosomes, *etc*. is shown on the top. **B.** Clustering dendrogram of cell types based on normalized nucleosome occupancy in the NDRs fails to reveal the neural progenitor–progeny relationships. TSS, transcription start site; NDR, nucleosome-depleted region.

Accurate nucleosome remodeling, especially around transcription start sites (TSSs), is critical to cell fate transition [18]. Therefore, we profiled nucleosome organization around TSSs. The result shows the typical arrangement at −1, NDR, +1, +2, +3 nucleosomes, *etc*. around TSSs in all four cell types. The nucleosome array in the regions downstream of TSSs gradually weakens as nucleosome positioning extends into the NDR borders to shorten the NDRs. The nucleosome array finally disappears when the NDRs are fully occupied by nucleosomes (Figure 2A). Further unsupervised hierarchical clustering analysis based on the nucleosome occupancy in the NDRs grouped the NSCs and the GNP cells together and left neurons and glia outside of the group (Figure 2B). The resultant clustering structure deviates from the lineage-commitment relationships between the four cell types. This suggests that nucleosome organization in the NDRs alone is not able to identify the unique and consistent difference between the cell types.

### H3K27me3 signal in the promotors reveals the lineage-commitment paths of NSCs

**Figure 3:**
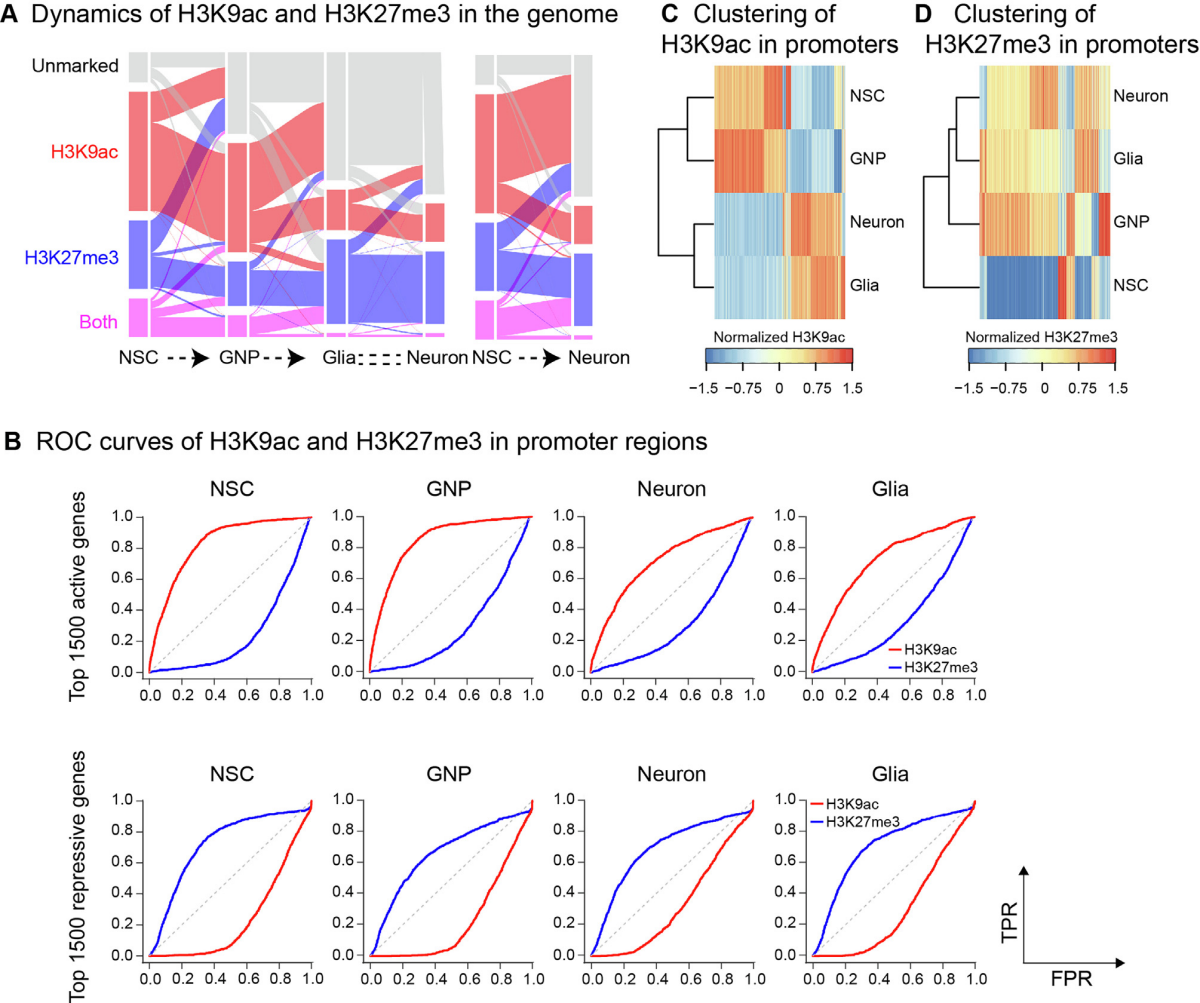
**HMs in the promoters are predictive for the differentiation potential of the neural progenitors** **A.** Alluvial plots showing the dynamics of H3K9ac and H3K27me3 in the genome. Regions having no H3K9ac or H3K27m3 found are labeled as unmarked, whereas regions with both H3K9ac and H3K27me3 found as labeled as both. **B.** ROC curves of H3K9ac and H3K27me3 in the promoter regions as predictors of gene activity using the top 1500 highly expressed genes as actual positives (top panels) and the bottom 1500 lowly expressed genes as actual positives (bottom panels). Unsupervised hierarchical clustering of normalized H3K9ac (**C**) and H3K27me3 (**D**) signals in the promoter regions showing the different progenitor–progeny relationships in the neuroglia differentiation. ROC, receiver operating characteristic; TPR, true positive rate; FPR, false positive rate.

We next examined HM changes in the promoters during the neuroglial development. Gene transcription is poised when the promoter is marked by both H3K4me3 and H3K27me3, *i.e.*, bivalent [19]. Bivalent promoters are rarely seen in *Drosophila* genome [20]. The low level of H3K9ac is important to the glial differentiation in *Drosophila* [15]. Therefore, we profiled H3K9ac and H3K27me3 in the *Drosophila* genome using ChromHMM [21] and classified the genome into four chromatin states: H3K9ac^+^, H3K27me3^+^, both (H3K9ac^+^/H3K27me3^+^), and unmarked. Almost half of the genomic regions are marked by H3K9ac in both NSCs and GNP cells, whereas only an insignificant fraction (14.9% and 15.7%) of the genome is marked by H3K9ac in neurons and glia (Figure 3A). Moreover, most of H3K9ac^+^ regions are maintained from NSCs to GNP cells. Although most of H3K9ac^+^, H3K27me3^+^ and H3K9ac^+^/H3K27me3^+^ regions in GNP cells are inherited from NSCs, approximately one third of H3K9ac^+^ and half of H3K27me3^+^ regions in NSCs become unmarked in GNP cells. This suggests that there exist characteristic chromatin states between NSCs and GNP cells, albeit both are pluripotent progenitors. In contrast, neurons and glia share similar genome-wide chromatin states. As expected, chromatin states are prominently different between the progenitor cells and the differentiated cells (Figure 3A).

The characteristic difference in chromatin states of the four cell types prompted us to examine that specific HM(s) could be used to construct the neuroglial development pathways. We first analyzed the correlation between HMs in the promoters and the gene activity. The results show both H3K9ac and H3K27me3 signals in the promoters are efficient indicators of gene activity (Figure 3B). Specifically, the H3K27me3 change in the promoters is inversely correlated with the change in gene expression from NSCs to GNP cells (Figure S1 and Figure S2A). Consistently, it has been reported that the signals of H3K9ac and H3K27me3 in the promoters are predictive markers for gene activity in the neuroectodermal differentiation of human ESCs [9].

We next classified the four neural cell types using the signals of H3K9ac and H3K27me3 in the promoters. The result shows that H3K9ac signal in the promoters separates the progenitor cells (NSCs and GNP cells) from the differentiated cells (neurons and glia) (Figure 3C). In contrast, H3K27me3 signal in the promoters constructs the lineage-commitment paths of NSCs (Figure 3D). Together, chromatin states in promoters are predictive for gene activity. Furthermore, H3K27me3 signal in the promoters can reveal the differentiation potential of progenitor cells.

### H3K27me3 signal in the enhancers reveals the differentiation potential of progenitor cells

**Figure 4:**
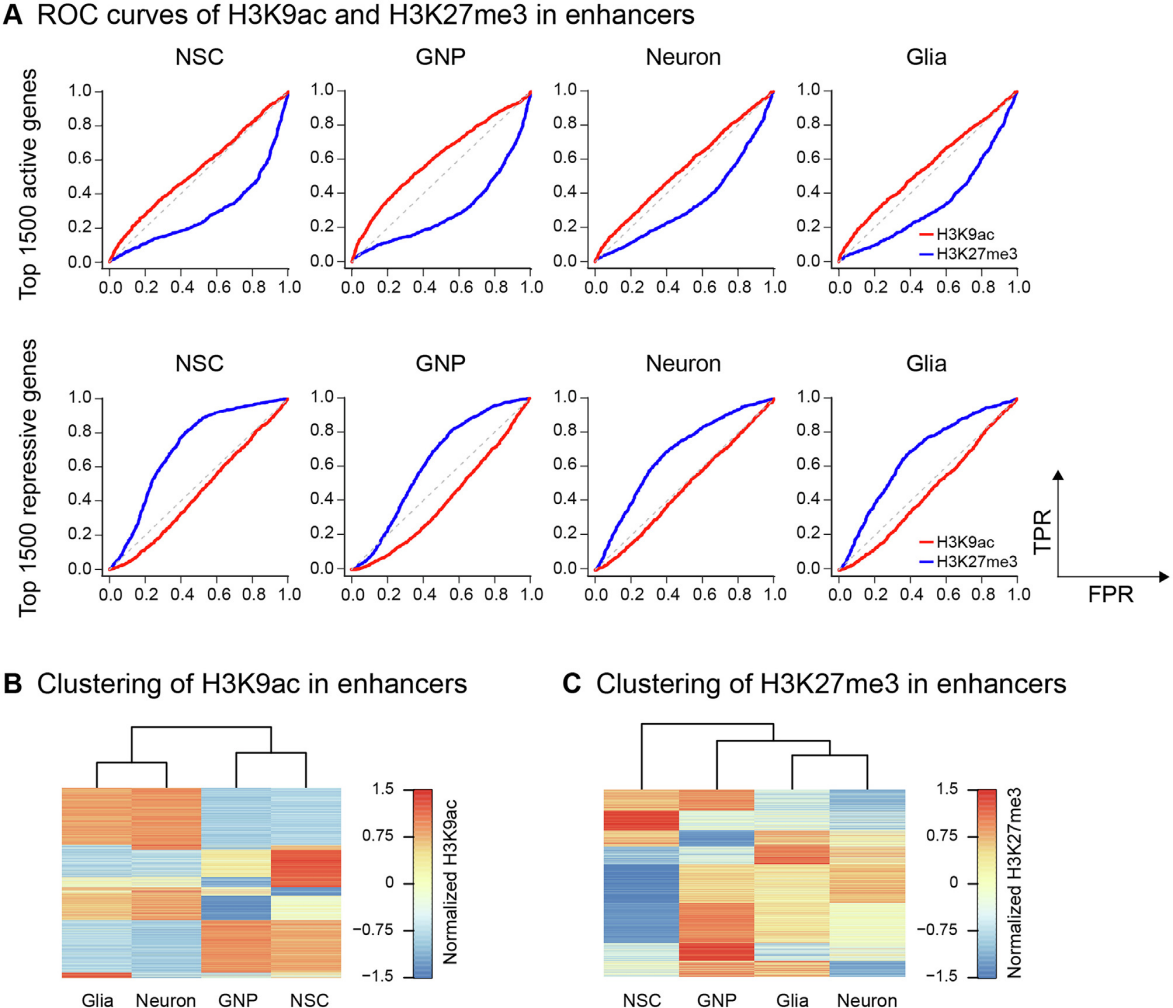
**H3K27me3 signal in the enhancers constructs the neural differentiation path** **A.** ROC curves of H3K9ac and H3K27me3 in the enhancers as predictors of gene activity using the top 1500 highly expressed genes as actual positives (top panels) and the bottom 1500 lowly expressed genes as actual positives (bottom panels). Unsupervised hierarchical clustering of normalized H3K9ac (**B**) and H3K27me3 (**C**) signal in the enhancers showing the differential grouping of lineage progenitors (NSC and GNP).

Enhancers are distal *cis* regulatory elements critical to establish and maintain cell identity [22], [23]. Since H3K4me1 is the predictive chromatin signature of enhancers [24], we identified enhancers by predicting H3K4me1 peaks using HOMER [25] (details in Methods). In addition, HMs in enhancers determine the chromatin state [23]. For example, enhancers with H3K27me3 are poised. Thus, we analyzed the correlation between HMs in enhancers and gene activity. The results show that H3K9ac signal in enhancers is not predictive for gene activity but can separate the progenitor cells from the differentiated cells (Figure 4A and B). However, H3K27me3 signal in enhancers is not only predictive for gene activity but also can construct the neuroglial development path (Figure 4A and C). Specifically, the H3K27me3 change in the enhancers is inversely correlated with the change in gene expression from NSCs to GNP cells (Figure S2B). This result agrees with the reported finding that enhancer establishment and its chromatin state can reveal the differentiation potential of progeny during hematopoiesis [11].

### H3K9ac signal in enhancers is associated with neuron- and glia-related functions

Although H3K9ac signal in promoters and enhancers fails to reveal the differentiation potential of progenitor cells (Figure 3C and Figure 4B), a low level of global H3K9ac is required for gliogenesis in *Drosophila* [15]. Conversely, we found that H3K9ac signal in promoters was increased in the glia-specific genes up-regulated during the glial differentiation in *Drosophila* [13]. Additionally, H3K9ac signal in promoters was increased during the neuronal differentiation in *Drosophila* [12]. This indicates that H3K9ac signal in promoters activates distinct sets of genes with neuron- and glia-related functions during their differentiation.

**Figure 5:**
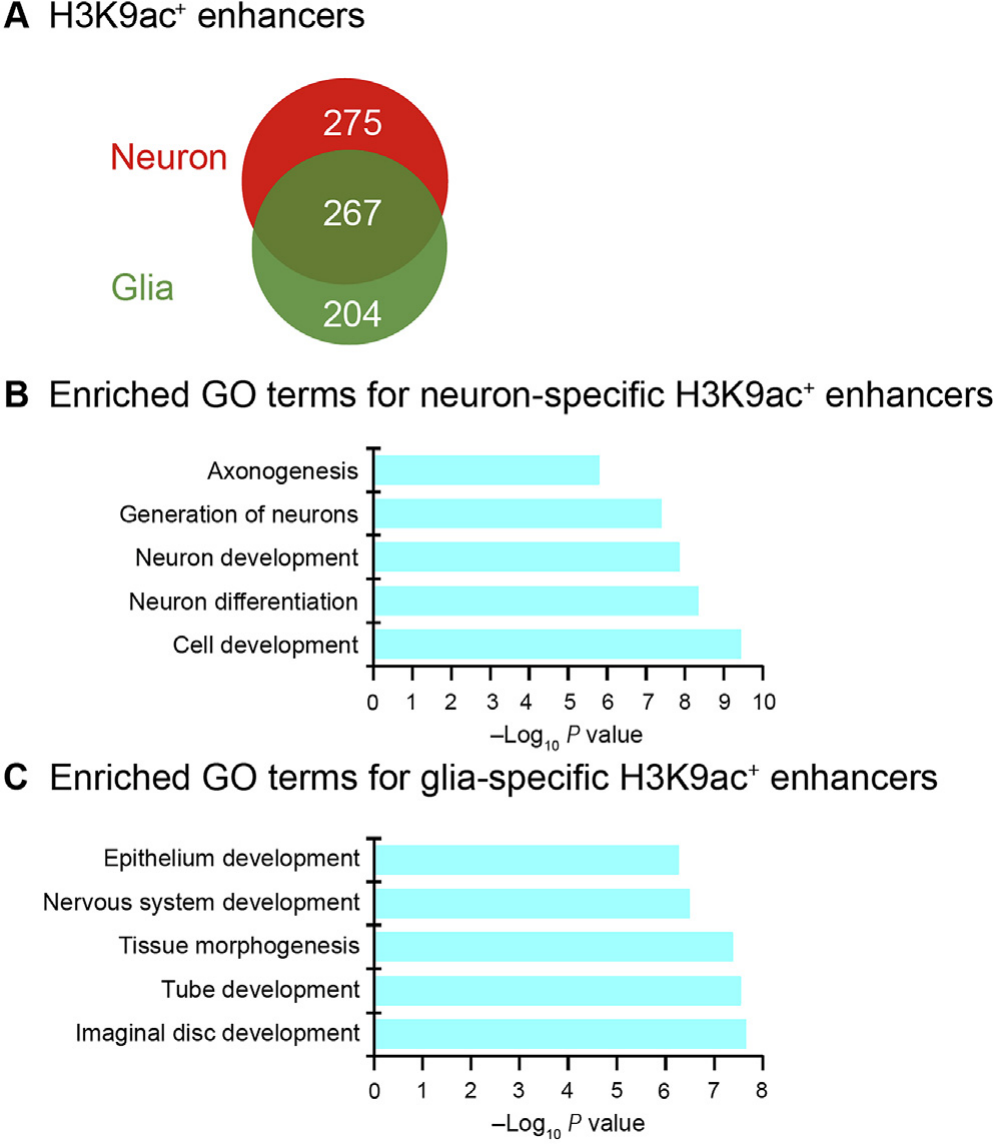
**H3K9ac signal in the enhancers is associated with neuron- and glia-related functions** **A.** Venn diagram showing the overlap between neuron- and glia-specific H3K9ac^+^ enhancers. **B.** Functional annotation of neuron-specific H3K9ac^+^ enhancers. **C.** Functional annotation of glia-specific H3K9ac^+^ enhancers.

Similarly, in order to understand the distinct functions of H3K9ac in enhancers in neurons and glia, we compared the neuron- and glia-specific H3K9ac^+^ enhancers and find that approximately half of H3K9ac^+^ enhancers are specific to neurons or glia (Figure 5A). We define the nearest gene of an enhancer as its target gene. Consequently, we obtained 92 target genes for the neuron-specific H3K9ac^+^ enhancers and 79 target genes for the glia-specific H3K9ac^+^ enhancers, respectively (Table S1), among which six genes were commonly found in both groups of enhancers. The gene ontology (GO) functional annotation shows that the neuron-specific target genes are enriched for generation of neurons, neuron development, neuron differentiation, *etc*. (Figure 5B), whereas glia-specific target genes are enriched for nervous system development, tissue morphogenesis, *etc*. (Figure 5C). This suggests that H3K9ac signal in enhancers activates the target genes with functions specific to neural cell subtypes.

Dynamics of HMs in promoters and enhancers have been reported to synergistically regulate gene activity in the cell fate transition [26]. We have also revealed that HM changes in promoters and enhancers synergistically regulate gene expression during the neuronal differentiation in *Drosophila* embryos [12]. Here, we find that HMs in promoters and enhancers are crucial for *Drosophila* neuroglial development. However, it is not clear whether there exists looping between enhancers and promoters for their synergistic regulation. Further study of chromatin interactions through chromatin conformation capture carbon copy (5C) or Hi-C technology could resolve this issue.

## Materials and methods

### Data collection

The high-throughput sequencing data of the four types of neural cells (NSCs, GNP cells, glia, and neurons) were from our recent work [12], [13]. In brief, four distinct *Drosophila* strains were generated for purification of these cells. NSCs and GNP cells were isolated from 5 to 7 h after egg laying (AEL) embryos (stage 11), whereas glia and neurons were isolated from 12 to 14 h AEL embryos (stages 15 and 16). The nuclei specific for the four types of neural cells were purified from the corresponding embryos using INTACT technology [27].

Nuclear RNA was extracted from nuclei that were isolated with the RNeasy Micro Kit (Catalog No. Y5-74004, Qiagen, Valencia, CA). Mononucleosomes were obtained from the nuclei by MNase digestion. Nucleosomes with specific HMs were further purified using the corresponding HM antibodies. The purified RNA, as well as nucleosomal DNA with and without HMs, was used to construct sequencing library with standard Illumina library prep protocols. The sequencing was performed on Illumina HiSeq2000 system with read length of 49 bp, single end. The sequencing datasets were deposited in the Gene Expression Omnibus database with the accession numbers GSE80458 and GSE83377.

### RNA-seq analysis

We mapped sequencing reads to the annotated *Drosophila* transcripts (FlyBase r5.43) using the tool Tophat (v1.3.1) with the default parameters [28], and retained the uniquely mapped reads to calculate gene expression levels using Cuffdiff (v1.3.0) [28]. We calculated and normalized gene expression levels as read per kilobase per million mapped reads (RPKM), and identified the differentially expressed genes (DEGs) between two samples with false discovery rate (FDR) < 0.05.

### MNase-seq analysis

We mapped the nucleosomal reads to the reference genome (dm3) of *Drosophila* using Bowtie with maximal two mismatches [29]. We next retained the uniquely mapped reads for nucleosome analysis. We then calculated nucleosomal read counts using a 10 bp bin in the 2 kb regions centered at TSS. We further calculated and normalized the read count in each bin as read per million mapped reads (RPM) that was represented in heatmaps. Regions ranging from 150 bp upstream to 50 bp downstream of TSS were defined as NDRs. Genes were ascendingly ordered by nucleosome occupancy in the NDRs.

### HM signal in promoters and enhancers

We mapped the HM reads to the reference genome (dm3) of *Drosophila* using Bowtie with maximal two mismatches [29], and retained the uniquely mapped reads for downstream analysis.

Regions ranging from 1 kb upstream to 1 kb downstream of TSS were defined as the promoters. We calculated and normalized HM read counts in the promoters as RPM in the same way of MNase-seq analysis mentioned above.

Enhancers were defined as H3K4me1 peaks predicted by HOMER [25] with a 1 kb sliding window and FDR of 0.001. H3K4me1 peaks overlapping with promoter regions were discarded. H3K9ac and H3K27me3 read counts in the enhancers were calculated and normalized as RPM in the same way as for the promoters.

### Clustering analysis

Unsupervised clustering of gene expression was conducted as following. First, only DEGs between any two samples were kept. Second, gene expression levels of all samples were normalized together using Z-score. Third, hierarchical clustering of the normalized gene expression was done by Euclidean distance on both samples and genes. Unsupervised clustering of nucleosome occupancy in the NDRs, as well as HM signal in promoters and enhancers, was done similarly as for the gene expression clustering.

### Receiver operating characteristic analysis of HMs in the proximal and the distal cis regulatory elements

Receiver operating characteristic (ROC) curve was applied to study which HM(s) in promoters could best classify gene activity. The top 1500 highly expressed genes were labeled as actual positives, with the remaining genes as actual negatives. H3K9ac signal in promoters was descendingly sorted and used as thresholds. We calculated the true positive rate (TPR) and false positive rate (FPR) for each threshold. Then, the ROC curve was created by plotting TPR against FPR.

The ROC curve for H3K27me3 signal in promoters was plotted in the same manner except that the bottom 1500 lowly expressed genes were defined as actual positives, with the remaining genes as actual negatives.

ROC analysis of HMs in enhancers was done similarly as for promoters except that data of HMs in enhancers were used.

### Dynamic changes of HMs in the genome

Chromatin states were identified and characterized using chromHMM (v1.14) [21] as follows: we transformed the Bowtie alignment files of H3K9ac and H3K27me3 into 200 bins using BinarizeBam function. We then trained the model with four emission states with LearnModel using default parameters. The genome of each cell type was then classified into four chromatin states: H3K9ac^+^, H3K27me3^+^, H3K27me3^+^/H3K9ac^+^ (termed as both) and none of the two HMs (termed as unmarked).

### Functional annotation of the target genes of enhancers

The nearest gene to an enhancer is defined as the target gene of the enhancer. Genes whose TSSs are more than 10 kb away from the proximal border of enhancers were discarded. The online tool DAVID (v6.8) was used to analyze GO enrichment [30]. Only the terms in biological process were considered and the top five significantly enriched terms were retained.

## Authors’ contributions

CJ conceived the study. YY did most of the experiments with the help of LG and YD. XC did most of data analyses with the help with JS, WJL, WL, and XZ. XC and CJ wrote the manuscript. All authors read and approved the final manuscript.

## Competing interests

The authors have declared no competing interests.

## Supplementary material

Supplementary Figure S1 **H3K27me3 signal in the promoters is negatively correlated with gene expression from NSCs to GNP cells** Gene expression levels are significantly decreased as H3K27me3 signals in the promoters are significantly increased (**A**). Contrarily, gene expression levels are significantly increased as H3K27me3 signals in the promoters are significantly decreased (**B**). ^***^*P* < 0.001, Wilcoxon’s rank sum test.

Supplementary Figure S2 **Changes in HMs and expression levels of representative genes from NSCs to GNP cells A.** Track view of expression levels and HM signals of gene Surf6. The increased H3K27me3 signals in the promoter (marked in the red box) are negatively correlated with the decreased expression level. In contrast, H3K9ac signals are not changed. **B.** Track view of expression levels and HM signals of gene CG30428. The increased H3K27me3 signals in the enhancer (marked in the red box) are negatively correlated with the decreased expression level. In contrast, H3K4me1 and H3K9ac signals are not significantly changed.
