# Supplementary material for: H3K27me3 Signal in the Cis Regulatory Elements Reveals the Differentiation Potential of Progenitors During Drosophila Neuroglial Development
Source: Genomics Proteomics Bioinformatics. 2019 Jun 11;17(3):297–304. doi: 10.1016/j.gpb.2018.12.009 (PMC6818177; doi:10.1016/j.gpb.2018.12.009)
Supplement: Supplementary Figure S1 — H3K27me3 signal in the promoters is negatively correlated with gene expression from NSCs to GNP cells Gene expression levels are significantly decreased as H3K27me3 signals in the promoters are significantly increased (A). Contrarily, gene expression levels are significantly increased as H3K27me3 signals in the promoters are significantly decreased (B). ***P < 0.001, Wilcoxon’s rank sum test. [file mmc1.pdf]

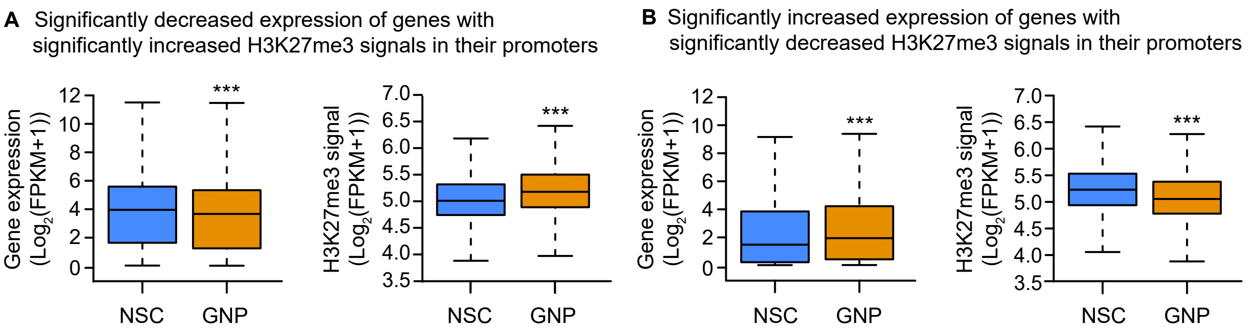
A Significantly decreased expression of genes with significantly increased H3K27me3 signals in their promoters
Gene expression (Log2(FPKM+1))
0
2
4
6
8
10
12
***
NSC
GNP
H3K27me3 signal (Log2(FPKM+1))
3.5
4.0
4.5
5.0
5.5
6.0
6.5
7.0
***
NSC
GNP
B Significantly increased expression of genes with significantly decreased H3K27me3 signals in their promoters
Gene expression (Log2(FPKM+1))
0
2
4
6
8
10
12
***
NSC
GNP
H3K27me3 signal (Log2(FPKM+1))
3.5
4.0
4.5
5.0
5.5
6.0
6.5
7.0
***
NSC
GNP
