# Supplementary figures and images for: H3K27me3 Signal in the Cis Regulatory Elements Reveals the Differentiation Potential of Progenitors During Drosophila Neuroglial Development
Source: Genomics Proteomics Bioinformatics. 2019 Jun 11;17(3):297–304. doi: 10.1016/j.gpb.2018.12.009 (PMC6818177; doi:10.1016/j.gpb.2018.12.009)

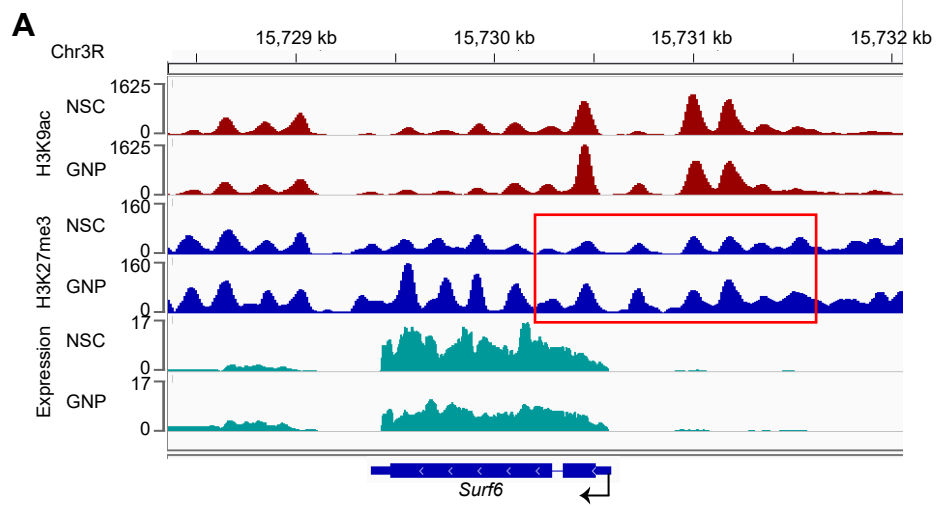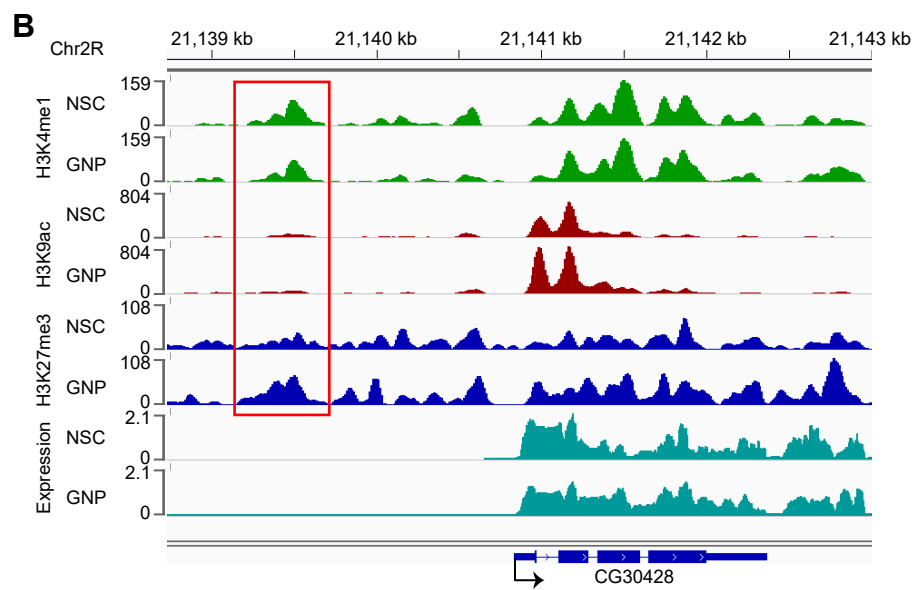

Supplement: Supplementary Figure S2 — Changes in HMs and expression levels of representative genes from NSCs to GNP cells A. Track view of expression levels and HM signals of gene Surf6. The increased H3K27me3 signals in the promoter (marked in the red box) are negatively correlated with the decreased expression level. In contrast, H3K9ac signals are not changed. B. Track view of expression levels and HM signals of gene CG30428. The increased H3K27me3 signals in the enhancer (marked in the red box) are negatively correlated with the decreased expression level. In contrast, H3K4me1 and H3K9ac signals are not significantly changed. [file mmc2.pdf]
